# Epidemiology of malaria in pregnant women attending antenatal consultation in Dschang, West Cameroon

**DOI:** 10.4102/jphia.v16i1.1437

**Published:** 2025-08-20

**Authors:** Calvin B. Ebai, Flore N. Ngoufo, Rene N. Teh, Jerline T.S. Kodjo, Eminline J. Muyang, Helen K. Kimbi

**Affiliations:** 1 Department of Medical Laboratory Science, Faculty of Health Sciences, University of Bamenda, Bamenda, Cameroon; 2 Division of Epidemiology and Biostatistics, Department of Global Health, Faculty of Medicine and Health Sciences, University of Stellenbosch, Cape Town, South Africa; 3 Department of Biomedical Science, Faculty of Health Sciences, University of Bamenda, Bamenda, Cameroon; 4 Department of Animal Biology and Conservation, Faculty of Science, University of Buea, Buea, Cameroon; 5 Department of Microbiology and Parasitology, Faculty of Science, University of Bamenda, Bamenda, Cameroon

**Keywords:** epidemiology, prevalence, malaria, pregnant women, risk factors, Dschang, Cameroon

## Abstract

**Background:**

Despite measures, malaria in pregnancy is still reported. It results in maternal illness, anaemia, low birth weight, preterm delivery and both maternal and foetal death.

**Aim:**

To determine the prevalence and density of malaria parasitaemia and identify the associated factors among pregnant women.

**Setting:**

This was a hospital-based study in two health facilities in Dschang, Western Cameroon.

**Methods:**

A cross-sectional study was conducted. A questionnaire was used to collect data on socio-demographics clinical manifestations, environmental factors and prevention measures used. Parasitological tests were carried out using thick and thin blood smears. Data were analysed using Statistical Package for Social Sciences (SPSS), version 22.0.

**Results:**

Out of the 314 participants, 46 (14.6%) were positive for malaria parasitaemia, and the only species identified was *Plasmodium falciparum*. A multinomial regression model showed that the presence of bushes around houses (odds ratio [OR] = 2.40, *p* = 0.03) exposes individuals to malaria parasite infection, while the presence of a ceiling (OR = 0.20, *p* < 0.01), taking intermittent preventive treatment for pregnant women (IPTp) (OR = 0.23, *p* < 0.01) and having window screens (OR = 0.14, *p* = 0.01) were protective. Geometric mean parasite density (GMPD) was highest among pregnant women in the second trimester (2190/*µ*L, *F* = 61.3, *p* = 0.016), those with more than three gravidities (1022/*µ*L, *F* = 66.28, *p* = 0.009), those who presented with sweating (1946/*µ*L, *F* = 272, *p* = 0.004) and, unexpectedly, those who were using long-lasting insecticide-treated bed nets (1536/*µ*L; *F* = 3.32, *p* < 0.001), compared with their corresponding counterparts.

**Conclusion:**

The prevalence and density of malaria parasite varied with demographics, pregnancy characteristics, clinical manifestations, quality of housing, environmental conditions and malaria prevention methods.

**Contribution:**

An update on malaria among pregnant women. Continuous sensitisation on prevention methods is necessary.

## Introduction

Malaria is an infectious disease caused by protozoan parasites of the *Plasmodium* species. It is transmitted by infected female *Anopheles* mosquitoes when they feed on human blood. The disease remains an important public health problem, particularly in sub-Saharan Africa, including Cameroon with significant economic burden.^1^ Cameroon is among the 15 countries with the highest malaria burden in the world with 3.0% of all global malaria cases and 1.9% of malaria deaths in 2023.^1^ Malaria in pregnancy is a severe condition usually leading to maternal illness, maternal anaemia, low birth weight, preterm delivery and both maternal and foetal death.^2,3^ The occurrence and severity of the disease in pregnancy may be influenced by factors related to socio-demographics, environment, pregnancy, treatment, as well as uptake of control measures.^4,5,6^ The Cameroon Government within the past decades has taken several measures to eliminate malaria and especially reduce the burden on pregnant women. These measures include free distribution of long-lasting insecticide-treated bed nets (LLINs), intermittent preventive treatment for pregnancy (IPTp) through free distribution of sulfadoxine pyrimethamine (SP) for prophylaxis, as well as health education during visit to antenatal clinic (ANC).^7^ These measures have been reported to reduce the prevalence of malaria in some settings.^8,9^ However, there are reports of poor and non-uptake of the prevention measures by some pregnant women,^8,10^ accounting for the occurrence of malaria in pregnancy reported in some of the recent studies.^9,11^ Many malaria cases in pregnancy are asymptomatic and if undetected and untreated may lead to severity in disease and sometimes irreversible outcomes for both mother and foetus. Women in their first pregnancies have been reported to be more vulnerable to *Plasmodium* parasite infection.^4,11^

Factors influencing the occurrence of malaria vary with geographical settings, and identifying the risk factors within a specific setting is crucial for designing intervention measures towards effective control of the disease. This study aims to determine the prevalence of malaria parasitaemia and to identify the risk factors among pregnant women attending ANC at the Dschang Regional Hospital Annexe and the Saint Vincent de Paul Hospital in Dschang, West Cameroon. The paucity of updated data on malaria in pregnancy in these area, as well as the necessity for continuous monitoring of disease occurrence make this study relevant. This may provide evidence for informed decision-making and support a possible review of policy towards more effective control of malaria among pregnant women.

## Research methods and design

### Study design

This was a hospital-based cross-sectional study conducted in the month of April 2024. A pretested structured questionnaire was used to collect data on demographics and predisposing factors. Blood samples were collected through finger prick to detect and quantify malaria parasite.

### Study setting

This study was carried out at the Dschang Regional Hospital Annexe and the Saint Vincent de Paul Hospital in Dschang. Dschang is the headquarters of the Menoua Division of the West Region of Cameroon. It is a cosmopolitan town with an estimated population of over 99 000 inhabitants. It is located in the western highlands of Cameroon at longitudes 10.036850 W to 10.084987 E and latitudes 5.470937 N to 5.422352 S, at an altitude of about 1380 m above the sea level. Dschang has two seasons like most parts of Cameroon. The rainy season spans from mid-March to mid-November, while the dry season lasts from mid-November to mid-March. Temperatures, during the rainy season, range from 16.1 °C to 26.7 °C, with August being the coldest month, with an average temperature of 18.1 °C. In the dry season, the temperatures range from 17 °C to 33 °C, with February being the hottest month, having an average temperature of 23 °C. The annual rainfall averages 2145 mm. The vegetation is between the savannah and the forest types covered with very dense vegetation. Dschang has both urban and rural settings. Commerce is a major economic activity alongside a very active educational sector. Educational institutions at all levels in both the state and private sectors are present; a good proportion of the population also practise farming. Dschang is endowed with both state-owned and privately owned health facilities with the Regional Hospital Annexe, the highest referral and most equipped in the division. The Saint Vincent de Paul Hospital is a faith-based health facility owned by the Catholic Mission. Both health facilities operate ANC on a daily basis with equipped laboratory services.

### Study population and sampling

Enrolment of participants was done in the antenatal units of the two health facilities. A convenience sampling technique was used to recruit pregnant women. Excluded from this study were women who were severely ill with chronic diseases like acquired immunodeficiency syndrome (AIDS) and cancer; those who had not lived in Dschang for up to two months, to avoid persons coming from outside the study area with infections; and those who were undergoing malaria treatment, in order to reduce the chances of false negative results. However, we did not find any pregnant women who were identified into any of these categories during the study. Using Cochran’s formula^12^ and the malaria prevalence of 10.14% in Cameroon reported by Nlinwe et al.,^9^ the calculated sample size was 366 participants.

### Data collection

#### Administration of questionnaire

A standardised, pretested questionnaire was used to collect data on demographics, such as age, sex, level of education and profession; predisposing factors, including clinical and environmental aspects; history of pregnancy and the use of malaria prevention measures. Pregnancy age was divided into three trimesters, following the WHO prescriptions (1st: 0–13 weeks, 2nd: 14–26 weeks and 3rd: 27–40 weeks).^13^ The questionnaire was administered in English, Pidgin and French, depending on the participant’s preference. This was done with the assistance of healthcare personnel (nurses and midwives) on duty, as the women took rounds during their ANC consultation.

#### Blood specimen collection and laboratory procedures

For detection and identification of *Plasmodium* species, capillary blood was collected from either the ring finger or the middle finger using a sterile lancet after disinfection with 70% ethyl alcohol. Excluding the first drop, three drops of blood were collected; two drops were placed on clean, grease-free microscope slides at separate points and used to prepare thick and thin blood smears. The third drop of blood was used to perform a white blood cell count using the improved Neubauer counting chamber, following standard procedures. The blood smears were air dried and the thin smear fixed with absolute methanol. Both smears were stained with 10% Giemsa stain for 10 min and examined under a light microscope (Olympus, New York, United States), using the oil immersion objective, following standard operating procedures. The thick blood smears were used for parasite detection and quantification using the formula:


$$\frac{\begin{array}{l} \text{Malaria parasitaemia }(\text{MP})=\text{Parasaites counted}\\ \text{ against WBC/counted}\\ \text{ WBC}*\text{WBC of the}\\ \text{ participant}, \end{array}}{}$$ [Eqn 1]

while the thin blood smears were used for species identification.^14^

### Data analysis

Data was entered in a Microsoft Excel spreadsheet and exported into the Statistical Package for Social Sciences (SPSS), version 22.0. Descriptive statistics were presented as frequencies and percentages. Fisher’s exact test and the Chi-square test were used to compare prevalence of malaria among groups; odds ratios (OR) were used to identify factors associated with malaria parasitaemia, while logistic regression model was used to determine risk factors. A one-way analysis of variance (ANOVA) test was used to compare geometric mean parasite densities (GMPDs) at 95% confidence interval (CI). The level of significance was set at *p* < 0.05.

### Ethical considerations

The ethical clearance for this study was obtained from the Institutional Review Board/Ethics Committee of the Faculty of Health Sciences, University of Bamenda (No. 2024/0343H/UBa/IRB of 05 April 2024). Administrative authorisation was obtained from the Director of the Dschang Regional Hospital Annexe as well as from the Director of Saint Vincent de Paul Hospital Dschang. Participants were informed about the objectives, procedures, benefits and inconveniences of the study. Those who agreed to take part in the study gave written informed consent. Confidentiality of participants was ensured by use of codes on all documents; no names were used. Participation in the study was completely free and voluntary; participants had the right to quit at any time without facing any consequences.

## Results

### Socio-demographic characteristics of the study participants

A total of 314 participants – 212 (64.3%) from the Regional Hospital Annexe and 102 (35.7%) from the Saint Vincent De Paul Hospital – were admitted into the study. The age range was from 17 years to 40 years, with the median age being 26 years. The age group 17–25 years was the most represented (*n* = 144, 45.9%). Most of the women were married (*n* = 191, 60.8%), 146 (46.5%) had attained secondary education, and 107 (34.1%) were in business (Table 1).

**TABLE 1:** Socio-demographic characteristics of the study population.

Characteristic	Category	Frequency or value	%
Age (years)	Range	17–40	-
	Median	26	-
	17–25	144	45.9
	26–30	99	31.5
	31–40	71	22.6
Marital status	Married	191	60.8
	Single	123	39.2
Level of education	No formal education	10	3.2
	Primary	26	8.3
	Secondary	146	46.5
	Tertiary	132	42.0
Occupation	Business	107	34.1
	Farmer	18	5.7
	Salary earner	45	14.3
	Housewife	60	19.1
	Student	84	26.8
Tribe	Bamileke	249	79.3
	Banwa	23	7.3
	Others†	42	13.4
Health facility	Saint Vincent de Paul Hospital	112	35.7
	Regional Hospital Annexe	202	64.3

**Total**	**-**	**314**	**100.0**

† , Participants from other tribes resident in the study area.

### Prevalence of malaria parasite

#### Prevalence of malaria parasite with respect to socio-demographic characteristics

Out of 314 participants, 46 (14.6%) were positive for malaria parasitaemia and the only species identified was *Plasmodium falciparum*. Comparing the prevalence of malaria parasitaemia between participants using Fisher’s exact test (FET), a significant statistical difference was observed with respect to the level of education (FET = 7.88, *p* = 0.04), with the highest prevalence detected among those with no formal education (30.0%). A similar observation was found with respect to the occupation, with the highest prevalence observed among farmers (*n* = 6, 33.3%; FET = 11.25, *p* = 0.02) (Table 2).

**TABLE 2:** Prevalence of malaria parasite with respect to socio-demographic characteristics.

Characteristic	Category	Frequency		Negative		Positive		FET	*p*
		*n*	%	*n*	%	*n*	%		
Age (years)	17–25	144	45.9	117	81.3	27	18.8	-	-
	26–30	99	31.5	90	90.9	9	9.1	4.40	0.11
	31–40	71	22.6	61	85.9	10	14.1	-	-
Marital status	Married	191	60.8	168	88.0	23	12.0	2.65	0.07
	Single	123	39.2	100	81.3	23	18.7	-	-
Level of education	No formal education	10	3.2	7	70.0	3	30.0	-	-
	Primary	26	8.3	20	76.9	6	23.1	7.88	0.04
	Secondary	146	46.5	121	82.9	25	17.1	-	-
	Tertiary	132	42.0	120	90.9	12	9.1	-	-
Occupation	Business	107	34.1	91	85.0	16	15.0	-	-
	Farmer	18	5.7	12	66.7	6	33.3	-	-
	Salary earner	45	14.3	44	97.8	1	2.2	11.99	0.01
	Housewife	60	19.1	49	81.7	11	18.3	-	-
	Student	84	26.8	72	85.7	12	14.3	-	-
Tribe	Bamileke	249	79.3	207	83.1	42	16.9	-	-
	Banwa	23	7.3	21	91.3	2	8.7	4.8	0.09
	Others†	42	13.4	40	95.2	2	4.8	-	-
Health facility	Saint Vincent de Paul Hospital	112	35.7	100	89.3	12	10.7	2.16	0.09
	Regional Hospital Annex	202	64.3	168	83.2	34	16.8	-	-

**Total**	**-**	**314**	**100**	**268**	**85.4**	**46**	**14.6**	**-**	**-**

FET, Fisher’s exact test.

† , Participants from other tribes resident in the study area.

### Factors associated with malaria parasitaemia

#### Pregnancy-related factors

Although the prevalence of malaria parasitaemia was highest among pregnant women in the first trimester (*n* = 11, 19.9%), there was no significant statistical difference (χ^2^ = 1.41, *p* = 0.49). With respect to gravidity, women at gravida 1 were most infected (*n* = 21, 17.4%), but the difference was not statistically significant (χ^2^ = 1.17, *p* = 0.56) (Table 3).

**TABLE 3:** Prevalence of malaria with respect to trimester of pregnancy and gravida.

Factor	Category	Tested		Negative		Positive		Level of significance	
		*n*	%	*n*	%	*n*	%	*χ* ^2^	*p*
Trimester	1st	58	18.5	47	81.0	11	19.0	-	-
	2nd	134	42.7	114	85.1	20	14.9	1.41	0.49
	3rd	122	38.9	107	87.7	15	12.3	-	-
Gravida†	Primigravida‡	121	38.5	100	82.6	21	17.4	-	-
	2–3	126	40.1	110	87.3	16	12.7	1.17	0.56
	> 3	67	21.3	58	88.6	9	13.4	-	-

*χ*^2^, Chi-square test.

† , Total number of pregnancies the participant had had;

‡ , First pregnancy.

#### Clinical and environmental factors

Participants presenting with the following clinical manifestations were at higher odds of being infected with malaria parasite than their corresponding counterparts: fever (OR = 6.34, 95% CI: 2.93–13.74, *p* < 0.01), headache (OR = 3.28, 95% CI: 1.72–8.23, *p* < 0.01) and muscle pain (OR = 4.82, 95% CI: 2.51–9.26, *p* < 0.01). Similarly, participants with the presence of bushes around their houses (OR = 3.10, 95% CI: 1.50–6.33, *p* = 0.001), those with mud house type (OR = 1.98, 95% CI: 1.02–3.83, *p* = 0.04) and those with holes on the walls of their houses (OR = 2.60, 95% CI: 1.26–5.25, *p* = 0.008) were at a higher risk of having malaria than their corresponding counterparts. The results also show that those who had a ceiling had a lower prevalence of malaria compared to those who did not (OR = 0.2, 95% CI: 0.11–0.44, *p* < 0.01), indicative of a protective effect of the ceiling against malaria infection (Table 4).

**TABLE 4:** Prevalence of malaria with respect to clinical and environmental factors.

Factor	Category	Tested		Negative		Positive		OR	95% CI	*p*
		*n*	%	*n*	%	*n*	%			
**Clinical factors**										
Fever	No	280	89.2	249	88.9	31	11.1	6.34	2.93–13.74	< 0.01
	Yes	34	10.8	19	55.9	15	44.1	-	-	-
Chills	No	309	98.4	263	85.1	46	14.9	0.85	0.81–0.89	0.35
	Yes	5	1.9	5	100	0	0.0	-	-	-
Sweats	No	258	82.2	222	86.0	36	14.9	1.34	0.62–2.9	0.45
	Yes	56	17.8	46	82.1	10	17.9	-	-	-
Headache	No	206	65.6	187	90.8	19	9.2	3.28	1.72–8.23	< 0.01
	Yes	108	34.4	81	75.0	27	25.0	-	-	-
Muscle pain	No	226	72.0	207	91.6	19	8.4	4.82	2.51–9.26	< 0.01
	Yes	88	28.0	61	69.3	27	30.7	-	-	-
Nausea	No	236	75.0	206	86.9	31	13.1	1.6	0.80–3.1	0.18
	Yes	78	25.0	63	80.8	15	19.2	-	-	-
Vomiting	No	291	92.7	249	85.6	42	14.4	1.25	0.40–3.85	0.70
	Yes	23	7.3	19	82.6	4	17.4	-	-	-
**Environmental factors**										
Bush around house	No	143	45	132	92.3	11	7.7	3.10	1.50–6.33	< 0.01
	Yes	171	54.5	136	79.5	35	20.5	-	-	-
Stagnant water around house	No	283	90.1	240	84.8	43	15.2	0.17	0.17–2.05	0.41
	Yes	31	9.9	28	90.3	3	9.7	-	-	-
House type†	Cement blocks	146	46.5	131	89.7	15	10.3	1.98	1.02–3.83	0.04
	Mud blocks	168	53.5	137	81.5	31	18.5	-	-	-
Holes on walls	No	261	83.1	229	87.7	32	12.3	2.60	1.26–5.25	0.008
	Yes	53	16.9	39	73.6	14	26.4	-	-	-
Have a ceiling	No	51	16.2	33	64.7	18	35.3	0.21	0.11–0.44	< 0.01
	Yes	263	83.8	235	89.4	28	10.6	-	-	-

Note: Tested: Number of participants tested for the variable.

OR, odds ratio; CI, confidence interval.

† , Material used to build the walls of the house.

#### Malaria prevention methods

It was observed that the prevalence of malaria parasite was lower among pregnant women who took IPTp than their counterparts who did not take (OR = 0.33, 95% CI: 0.16–0.70, *p* = 0.002). In the same light, a lower prevalence of malaria parasite was observed among participants who had window screens than those who did not have (OR = 0.84, 95% CI: 0.80–0.88, *p* = 0.02). This is indicative of a protective effect of both methods (Table 5).

**TABLE 5:** Prevention measures associated with malaria parasite prevalence.

Prevention method	Category	Tested		Negative		Positive		OR	95% CI	*p*
		*n*	%	*n*	%	*n*	%			
Taken IPTp	No	173	55.1	138	79.8	35	20.2	0.33	0.16–0.70	0.002
	Yes	141	44.9	130	92.2	11	7.8	-	-	-
Have LLINs	No	70	22.3	61	87.1	9	12.9	1.21	0.55–2.65	0.63
	Yes	244	77.7	207	84.8	37	15.2	-	-	-
Use of LLINs	No	142	45.2	118	83.1	24	16.9	0.72	0.38–1.35	0.30
	Yes	172	54.8	150	87.2	22	12.8	-	-	-
Have window screens	No	287	91.4	241	84.0	46	16.0	0.84	0.80– 0.88	0.02
	Yes	27	8.6	27	100	0	0.0	-	-	-
Use of mosquito coils	No	298	94.9	254	85.2	44	14.8	0.82	0.18–3.75	0.80
	Yes	16	5.1	14	87.5	2	12.5	-	-	-
Use of insecticidal spray	No	270	86.0	227	84.1	43	15.9	0.38	0.11–1.30	0.11
	Yes	44	14.0	41	93.2	3	6.8	-	-	-
Use of mosquito repellent	No	305	97.1	259	84.9	46	15.1	0.85	0.81–0.89	0.20
	Yes	9	2.9	9	100	0	0.00	-	-	-

Note: Tested: Number of participants tested for the variable.

OR, odds ratio; CI, confidence interval; IPTp, intermittent preventive treatment for pregnancy; LLIN, long-lasting insecticide-treated bed net.

### Determination of the risk factors of malaria parasitaemia

From the results of a multiple logistic regression model done using the environmental and prevention methods that were associated with malaria parasitaemia (*p* < 0.05), it was observed that the presence of bushes around the house (OR = 2.40, *p* = 0.03) exposes to malaria parasite infection, while the presence of a ceiling (OR = 0.20, *p* < 0.01), taking IPTp (OR = 0.23, *p* < 0.01) and having window screens (OR = 0.14, *p* = 0.01) were protective against malaria parasite infection (Table 6).

**TABLE 6:** Multinomial regression model for risk factors of malaria parasitaemia.

Factor	Unadjusted results			Adjusted results		
	cOR	95% CI	*p*	OR	95% CI	*p*
Bush around house	3.10	1.50–6.33	< 0.01	2.40	1.07–5.41	0.03
House type	1.98	1.02–3.83	0.04	0.97	1.1–5.41	0.93
Holes on walls of house	2.60	1.26–5.25	0.008	1.26	0.51–3.1	0.62
Have ceiling	0.21	0.11–0.44	< 0.01	0.20	0.08–0.44	< 0.01
Taken IPTp	0.33	0.16–0.70	0.002	0.23	0.10–0.53	< 0.01
Have window screens	0.84	0.80–0.88	0.02	0.14	0.01–2.1	0.01

cOR, crude (unadjusted) odds ratios; aOR, adjusted odds ratios; CI, confidence interval; IPTp, intermittent preventive treatment for pregnancy.

### Variation of parasite density with pregnancy-related factors, clinical manifestations and prevention methods

Assessing the variation of parasite density using one-way ANOVA, it was observed that there was a significant statistical difference in GMPDs with respect to trimester of pregnancy (*F* = 61.3; *p* = 0.016); with the second trimester having the highest (2191/*µ*L). A similar observation was obtained with respect to gravidity (*F* = 66.28; *p* = 0.009) with participants who had more than three pregnancies having the highest GMPD (1023/*µ*L). Participants manifesting with sweating (1242/*µ*L; *F* = 272; *p* = 0.004) and, surprisingly, those who used LLINs (1536/*µ*L; *F* = 175.3; *p* < 0.001) had higher GMPDs than their corresponding counterparts (Table 7).

**TABLE 7:** Comparing parasite density with respect to trimesters of pregnancy, clinical manifestations and malaria prevention methods.

Variable	Category	GMPD/*µ*L	s.e.	*F*-test	*p*
Trimester	1st	624.1	163.8	61.30	0.016
	2nd	2191	872	-	-
	3rd	1725	530	-	-
Fever	No	673	190	0.73	0.670
	Yes	1577	811	-	-
Gravida†	Primigravida‡	632	192	66.28	0.009
	2–3	357	199	-	-
	> 3	1023	436	-	-
Sweating	No	621	207	272.00	0.004
	Yes	1242	785	-	-
Headache	No	1759	1215	4.30	0.200
	Yes	781	275	-	-
Nausea	No	1414	413	0.35	0.870
	Yes	1160	360	-	-
Use of LLINs	No	720	250	175.30	< 0.001
	Yes	1536	485	-	-

GMPD, geometric mean parasite density; s.e., standard error; LLIN, long-lasting insecticide-treated bed nets.

† , Total number of pregnancies the participant had had;

‡ , First pregnancy.

## Discussion

Given that malaria continues to be a public health problem despite the continuous efforts by several stakeholders, vulnerable groups such as pregnant women continue to remain significantly affected by the disease. This study was carried out to assess the prevalence and risk factors of malaria parasitaemia in pregnant women. The prevalence of malaria parasite in this study was 14.6%. This figure of prevalence is higher than 5.06% reported in 2021 in Foumban, a district in the same region,^15^ as well as the 10.14% prevalence reported in 2022 in Foumbot, also within the same region and in Bamenda, which has similar environmental factors like the study site.^9^ These discrepancies could be because of variabilities in the implementation of control measures between health facilities, individual factors like immune status, and the differences in the demographic characteristics of the study populations. However, the prevalence of malaria parasitaemia found in this study is lower than the 39.6% reported by Elime et al.^16^ in Ndop, Cameroon; the 17.8% reported by Jugha et al. in the Mount Cameroon Region,^17^ and the 20.8% reported by Almaw et al. in Ethiopia.^18^ This could be because of an improvement in the overall implementation of preventive measures against malaria.

With respect to the levels of education, pregnant women with no formal education were most infected. This is most likely a consequence of lack of knowledge about the disease, and also the poor understanding of the importance and use of prevention measures. There is evidence that maternal education is a cost-effective intervention to reduce malaria infection.^19^ Concerning the occupation, farmers were most infected. This is likely because of their exposure to sites closer to vector hideouts and breeding sites, but also could be because of pig-rearing, which is common in the area, because mosquitoes are attracted to the animals for blood meal.^20^ Except for the levels of education and the occupation, there was no association observed between malaria parasitaemia and all other socio-demographic characteristics, including health facility. This similarity in prevalence could be an indication of the general efforts made by the government and other stakeholders to fight against malaria, especially among pregnant women and children below five years. Earlier studies have, however, shown associations between malaria parasitaemia and socio-demographic factors.^21,22^ The gaps may have been closed over time by a scale-up in prevention measures.

No significant statistical difference in prevalence was observed with respect to trimester and gravida. This could also be because of a scale-up in prevention measures. However, the highest prevalence was observed among the women in their first trimester and the primigravida. Similar results have been reported in other studies.^4,11^ This could be because of their lower levels of adaptive immunity.

Fever, headache and muscle pain were found to be associated with malaria parasitaemia in the studied participants. Malaria is characterised by sequential rupture of infected red blood cells (RBCs).^23^ During the rupture of RBCs, trophozoites and malaria pigment haemozoin are released into the blood circulation causing an immune response that results in synchronised bouts of fever corresponding to the moments of rupture.^24^ Prior to the occurrence of fever, there is usually a sensation of cold, which results in shivering to generate heat. This also results in an increase in the body temperature.^25^ Concerning headache, infected RBCs develop adhesiveness to both infected and non-infected RBCs; this results in sequestration of RBCs in the blood vessels, sometimes blocking microcirculation, including those in the brain and the skeletal muscles,^26^ which leads to hypoxia and impairment of tissue function. Also, there is the release of inflammatory cytokines because of the presence of malaria parasites, which stimulate the pain receptors in the tissues.^27^ These two processes could be a possible explanation for the headache and muscle pain observed in the malaria-infected participants in this study.

Furthermore, the presence of bushes around houses, houses fabricated with mud, the presence of holes on walls and the absence of a ceiling were associated with malaria parasitaemia. This confirms the findings in other studies too.^28^ Bushes around the house provide hideouts for the mosquito vector, thereby bringing them closer to the human host for infection. Holes on the walls are common in houses constructed with mud, and together with the absence of a ceiling they provide entry of mosquitoes *ad libitum*, thereby increasing the chances of malaria parasite transmission to the occupants.

Malaria prevention measures remain the key to the elimination or eradication of the disease. In this study, participants who failed to take IPTp (SP) were at higher risk of having malaria. The different components of sulfadoxine pyrimethamine (SP) are folate inhibitors. Sulfadoxine inhibits the activity of dihydropteroate synthase, an enzyme that catalyses the condensation of *para*-aminobenzoic acid (PABA) with 6-hydroxymethyl-7,8-dihydropterin pyrophosphate to form 7,8-dihydropteroate, a crucial step in the biosynthesis of folate, an essential molecule for the cell growth and division in parasites and other microorganisms.^29^ Pyrimethamine inhibits dihydrofolate reductase (DHFR), which plays a crucial role as an essential enzyme in the folate pathway, necessary for the synthesis of nucleotides required for deoxyribonucleic acid (DNA) replication and cell division. So, inhibiting DHFR disrupts the parasite’s ability to produce vital building blocks for its growth and survival,^29^ thus limiting the chances of establishing the disease.

Participants who did not have window screens were also at risk of infection. Window screens reduce the chances of the vector getting into the house to bite and transmit the parasite. The multiple logistic regression model confirmed the presence of bush around houses, the non-uptake of IPTp, the absence of a ceiling and window screens as the risk factors for malaria in the study population. Effective use of preventive measures has been reported to be protective against malaria transmission in several studies.^9,28^

Assessing the variation of GMPDs revealed significant statistical differences with respect to trimester of pregnancy, gravida, sweating and the use of LLINs. The highest values were found among participants in the second trimester, while the lowest values were observed in the first trimester. Inasmuch as the prevalence of malaria parasitaemia was higher among women in the first trimester, there is a possibility that improved immunity in preparation for pregnancy provides a general non-specific defence against infectious agents, including malaria parasite, as reported in some studies.^30,31^

A similar phenomenon was observed when comparing GMPDs with respect to gravida. Participants with more than three pregnancies had higher parasite densities than their counterparts with lesser pregnancies. These findings are unexpected given that reports have shown that women with higher gravida have greater specific immunity to malaria parasite and so will more likely eliminate malaria parasites than pregnant women with lower gravida.^32^ However, the results obtained here could be because of variabilities in the immunity of the individuals. Similar results were obtained in the study by Emmanuel et al.^33^ in Nigeria.

Higher GMPDs were also recorded in participants who presented with sweating compared to their counterparts. In a classical malaria bout, following the hot stage, there is usually profuse sweating, which is then followed by a reduction or drop in temperature and exhaustion, so this confirms sweating as a typical manifestation of malaria. Geometric mean parasite density was unexpectedly higher in participants who reported using mosquito bed nets. There could be a possible neglect on the part of these individuals, who may believe that because they slept under nets, they are not exposed to malaria, while failing to consider exposure when out of the net. This may expose them to multiple mosquito bites that could result in higher parasite densities in their blood.

### Limitation

This study did not ensure species confirmation of malaria parasite by polymerase chain reaction (PCR).

## Conclusion

The prevalence of malaria parasitaemia was associated with some demographic factors which included the levels of education and the occupation, as well as clinical factors, including fever, headache and muscle pain. The presence of bushes around houses, holes on walls, the absence of ceiling and non-uptake of IPTp were the risk factors for malaria. Geometric mean parasite densities varied significantly with pregnancy trimester, gravida, presence of profuse sweating and the use of LLINs. Some clinical signs may serve as predictors of malaria infection during pregnancy. Continuous sensitisation of pregnant women on the consistent use of prevention methods is essential.
